# Measuring Biases of Visual Attention: A Comparison of Four Tasks

**DOI:** 10.3390/bs10010028

**Published:** 2020-01-06

**Authors:** Ólafía Sigurjónsdóttir, Andri S. Bjornsson, Inga D. Wessmann, Árni Kristjánsson

**Affiliations:** 1 Faculty of Psychology, University of Iceland, Nýji Garður, 101 Reykjavík, Icelandasb@hi.is (A.S.B.); idw@hi.is (I.D.W.); 2 School of Psychology, National Research University Higher School of Economics, 101000 Moscow, Russia

**Keywords:** visual attention, emotional stimuli, anxiety, attentional bias, dot-probe task, attentional blink

## Abstract

Attention biases to stimuli with emotional content may play a role in the development and maintenance of anxiety disorders. The most commonly used tasks in measuring and treating such biases, the dot-probe and spatial cueing tasks, have yielded mixed results, however. We assessed the sensitivity of four visual attention tasks (dot-probe, spatial cueing, visual search with irrelevant distractor and attentional blink tasks) to differences in attentional processing between threatening and neutral faces in 33 outpatients with a primary diagnosis of social anxiety disorder (SAD) and 26 healthy controls. The dot-probe and cueing tasks revealed no differential processing of neutral and threatening faces between the SAD and control groups. The irrelevant distractor task showed some sensitivity to differential processing for the SAD group, but the attentional blink task was uniquely sensitive to such differences in both groups, and revealed processing differences between the SAD and control groups. The attentional blink task also revealed interesting temporal dynamics of attentional processing of emotional stimuli and may provide a uniquely nuanced picture of attentional response to emotional stimuli. Our results therefore suggest that the attentional blink task is more suitable for measuring preferential attending to emotional stimuli and treating dysfunctional attention patterns than the more commonly used dot-probe and cueing tasks.

## 1. Introduction

Selective attention allows us to choose the stimuli that are most relevant to current behavior from the vast barrage of visual information hitting our retinas at any given time. Certain types of stimuli seem to be preferentially attended to or can even capture our attention in a reflexive manner [1]. But attention can also be preferentially drawn to stimuli based on their meaning. The emotional content of stimuli can change how stimuli, that are otherwise similar, are attended to [2,3,4], such as whether faces have an emotional expression or not [5].

How people attend to stimuli with emotional content varies and strong attentional biases to emotional stimuli may fuel maladaptive attentional processing [6,7,8,9] which may play a role in the development and maintenance of anxiety disorders. A number of studies have suggested that anxious individuals selectively attend to threatening stimuli and do so to a greater degree than non-anxious individuals (see e.g., [8,10]). Such attention bias has been thought to involve either facilitated attention to threat or difficulty disengaging from it, and treatment programs aimed at correcting these biases have been developed [11,12,13,14].

The strength of any evidence for such claims, and the efficacy of such programs is, however, constrained by the quality of the tools used to measure potential biases. These tools need to be valid and reliable, but tasks commonly used for this purpose, the *dot-probe* [15] and *spatial cueing* tasks [16,17], have yielded mixed results in both measuring and modifying bias [13,18]. One potential problem is that these tasks provide only a snapshot of a dynamic process that unfolds over time. Tasks that capture these temporal dynamics may therefore be better-suited to measuring attentional bias.

We recently compared how well the dot-probe and cueing tasks measure potential processing differences between threatening and neutral stimuli, with two other tasks commonly used in contemporary studies of visual attention: *visual search with irrelevant distractors* [1] and *the attentional blink* task [19,20]. Surprisingly, the dot-probe and cueing tasks did not reveal any processing differences between threatening and neutral faces, while the attentional blink task proved to be highly sensitive to processing differences between these stimuli, also revealing interesting temporal dynamics [9]. The study was limited to a non-clinical student sample, however, and to assess any role of processing differences in triggering and/or maintaining anxiety, it is important to assess how accurately these tasks measure potential biases in clinical samples. Here we assessed these four tasks on individuals with social anxiety disorder (SAD) as a primary diagnosis, comparing their performance to healthy control participants. A valid and reliable task used to assess such processing differences should, firstly, detect differences in attentional processing of threatening and neutral stimuli, and secondly, differentiate between a clinical group and a control group without any anxiety. By assessing these tasks, our aim was to gain insights into attentional bias and its role in social anxiety disorder, and more generally, into the nature of attentional orienting to emotional stimuli.

## 2. Methods

### 2.1. Ethics Statement

All participants gave their informed consent for inclusion before they participated in the study. The study was conducted in accordance with the Declaration of Helsinki, and the experimental protocol was approved by the Icelandic Bioethics Committee (Permissions numbers 13-128 and 15-225).

### 2.2. Participants

The SAD group (n = 33) consisted of patients with SAD as their primary diagnosis seeking treatment at an outpatient anxiety treatment center. Exclusion criteria were current psychosis, mania or active suicidal ideation. The control group (n = 26) was selected from a community sample to match the SAD group on demographic variables, exclusion criteria being a current diagnosis of a psychiatric disorder and active suicidal ideation.

In the SAD group, 27% had current comorbid major depressive disorder, 15% had body dysmorphic disorder, 9% had panic disorder with agoraphobia, 6% had obsessive compulsive disorder, 6% had post-traumatic stress disorder and 6% had generalized anxiety disorder. Table 1 shows the key characteristics of the two samples.

Participants’ visual acuity was normal or corrected-to-normal. The groups did not differ in age or gender. Individuals in the SAD group reported more depression symptoms, greater severity of social anxiety, and lower quality of life.

### 2.3. Clinician Administered Interviews

To assess the clinical status of the participants, the measures listed below were used.

**Mini International Neuropsychiatric Interview**. The MINI [21] is a semi-structured interview assessing Axis I disorders as in *DSM-IV*. The MINI has high reliability and validity in relation to the Structured Clinical Interview for DSM-IV (SCID-IV) with inter-rater reliability ranging from 0.89 to 1.0 [21,22]. The Icelandic translation of the MINI has good convergent validity with self-report measures of depression and anxiety symptoms [23]. Inter-rater reliability was high both in the control group where agreement between raters was 100% for all disorders, and in the SAD group where it ranged from 90.9% to 100%.

**The Body Dysmorphic Disorder Diagnostic Module (BDD-DM)**. BDD-DM [24] is a brief semi-structured diagnostic interview for body dysmorphic disorder (BDD). In this study, the Icelandic version of the BDD-DM was used. The inter-rater reliability was high both for the control group where 87.5% agreement was for lifetime BDD and 100% for current BDD, and for the SAD group where 100% agreement was for lifetime BDD and 90.9% for current BDD.

**Liebowitz Social Anxiety Scale (LSAS)**. LSAS [25] is a semi-structured clinical rating scale designed to measure anxiety and avoidance in 24 social situations. Items are rated on a 0–3 point scale and the total score is comprised of the anxiety and avoidance subscales which give a measure of symptom severity for SAD. In this study, the Icelandic version was used. The internal consistency was good for both SAD (α = 0.82–0.94) and control groups (α = 0.77–0.90). Scores on the LSAS predicted a SAD diagnosis on the MINI. Inter-rater reliability was high on both subscales (i.e., the intraclass correlation coefficient [ICC] = 1.00 and 0.90 for anxiety and ICC = 0.91 and 0.94 for avoidance) as well as for the total score both for the control group (i.e., ICC = 0.98) and the SAD group (ICC = 0.92).

### 2.4. Self-Report Measures

**The Patient Health Questionnaire-9 (PHQ-9)**. The PHQ-9 [26] measures the severity of depressive symptoms during the past 2 weeks using 9 items on a 4 point Likert scale. PHQ-9 has high internal reliability and good test-retest reliability [26]. The Icelandic version had good internal consistency (α = 0.9) in this study.

**Quality of Life Scale (QOLS)**. The QOLS [27] measures quality of life on a seven point Likert scale ranging from 1 (terrible) to 7 (delighted). The Icelandic version of QOLS has been shown to have good internal (α = 0.89) and acceptable test-retest reliability (*r* = 0.72) [28]. In the current study the QOLS had an acceptable internal consistency (α = 0.72).

### 2.5. Administration of Clinical Tests and Interviews

Participants in the SAD group were recruited with advertisements seeking people aged 18–65 with social anxiety on social media and at an outpatient anxiety center. Control participants were recruited with advertisements on social media looking for people aged 18–65 not suffering from anxiety or depression. Potential participants for the control group were screened via email to match the SAD group on age and gender. All participants gave their informed consent. Trained assessors, licensed psychologists and graduate students in clinical psychology, evaluated all participants with the MINI, LSAS and BDD-DM interviews. Participants in the control group that met criteria for a psychiatric disorder were excluded from the study and referred to appropriate treatment. A licensed clinical psychologist supervised all assessments in weekly meetings where a consensus was reached regarding diagnoses.

### 2.6. Equipment

Experimental displays were programmed in C and presented on a 75-Hz CRT controlled by a 400-MHz G4 Apple computer. 

### 2.7. Visual Attention Tasks

**Figure 1:**
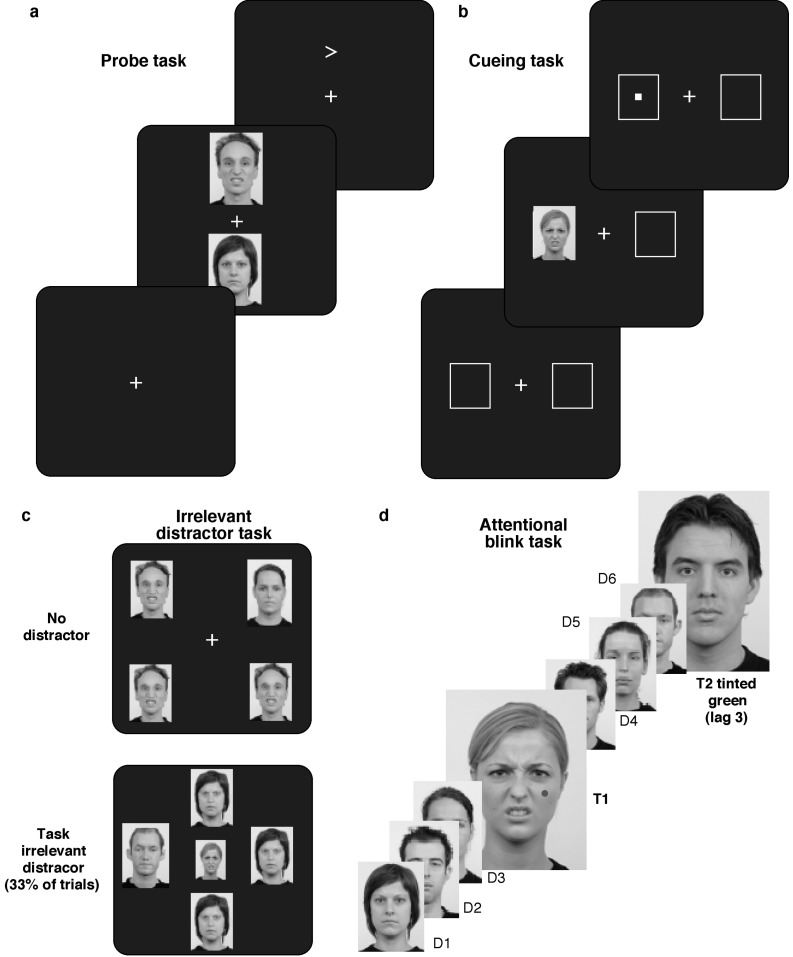
Experimental paradigms. (**a**) *Dot-probe task*: Two faces were presented for 146 ms followed by an arrow behind one of the faces (**b**) *Cueing task*: A face cue was presented for 146 milliseconds, immediately followed by the target at either the cued or uncued location. (**c**) *Irrelevant distractor task*: Participants searched for the odd face out and judged its gender while on 33% of trials a task-irrelevant face appeared at screen center (**d**) *Attentional blink task*: Participants had to detect the target that had a dot on either cheek (target 1, T1), and report the dot location and then judge the gender of the green tinted face (target 2, T2) that appeared 1–8 presentations later (T1 and T2 are inflated in size for demonstrative purposes only—all the images were of the same size in the experiment).

The stimuli in all the tasks were greyscale facial images of 39 young adults (20 males) from the *Radboud Faces Database* [29] with neutral or disgust expressions (threatening stimuli). All tasks started with the presentation of a central white fixation cross on a black background. The stimuli then appeared following a variable interval (1100 to 1500 ms, randomly determined for each trial (see Figure 1).

**Dot-probe task**. Two facial images (5.24° × 5.71°) of the same individual, one with a neutral expression and the other one a threatening expression appeared for 146 ms 3.5° above and below fixation. A white arrowhead (each line 30 arc min) followed, presented either where the neutral or threatening face had appeared. Participants judged (by keypress) whether the arrowhead pointed left or right. We measured whether response times changed by whether the arrow appeared behind a threatening or neutral face.

**Cueing task**. Two white frames (4.95° × 4.95°) appeared left and right of fixation (center: 4.5° from fixation). The cue, a neutral or threatening face, appeared for 146 ms 1100 to 1500 ms later (randomly determined) followed immediately by a small white square (30° × 30° arc min) either in the cue frame (valid cue) or the opposite frame (invalid cue; Figure 1b). Participants judged whether the square appeared on the left or right. We assessed whether performance differed by whether the cue was threatening or neutral.

**Visual search with irrelevant distractor**. Following fixation, four faces (5.71° × 4.57°) appeared. Three were of the same individual with one image of a member of the opposite gender. Participants had to find the odd-face-out and judge its gender (by keypress). The expression on each face was random. On 1/3 of trials, an irrelevant face (4.38° × 3.91°) with a random expression, which participants were instructed to ignore (random expression), appeared at center. We assessed slowing effects from the irrelevant distractor and whether they differed by its facial expression.

**Attentional blink task**. On each trial, a stream of 30 faces (5.24° × 5.90°) appeared at the center of the screen for 67 ms with a 40 ms blank screen in between. Two targets were embedded in the stream, T1 marked by a dot (0.19°) on the left or right cheek (randomly face number 5–15 in the stream) and T2 distinguished by green tint (face 8–18 following T1). The distractors were faces of different individuals presented in greyscale (either all neutral or all threatening). Participants judged by keypress whether the dot was on the left or right cheek on T1 and whether T2 was male or female. We assessed whether T2 detection would depend on the facial expression of T1 and/or T2.

### 2.8. Procedure

Participants attended two sessions, one two-hour session where they completed the three interviews and answered self-report measures. In the second session (~45–50 min), they completed the four computerized tasks in counterbalanced order: *Dot-probe task* (2 × 100 trials), *spatial cueing* (2 × 100 trials), *irrelevant distractor* (2 × 100 trials) and *attentional blink* (3 × 50 trials).

### 2.9. Data Analyses

For the dot-probe, cueing and irrelevant distractor tasks, trials with response times +/− 3 SDs for each participant (2% of the responses) were excluded, along with error trials for the dot-probe (4%), cueing (2%) and irrelevant distractor tasks (8.5%). Data were missing for six participants in the SAD group on one or more of the tasks due to corrupted data files or noncompletion. SAD group size for each task therefore varied from 29 to 32 participants. We used mixed-effects regression to analyze the data. The reported parameter estimates are from the best fitting model in each case (details in Section 6).

Mixed effect regression models were used to analyze the results. Mixed effect regression models include random effects to model between-participant heterogeneity in intercepts and slopes, taking the lack of independence of the repeated measurements into account and allowing explicit modeling of effects of different conditions (see e.g., [30]).

Three models were built for each task:

1. SAD model: Sensitivity to differences in attentional processing of threatening and neutral faces in the SAD group
2. Control model: Sensitivity to differences in attentional processing of threatening and neutral faces in the control group
3. Between-group model: Sensitivity to differences in anxious (SAD) and healthy (control) attentional processing of threatening and neutral faces.

The best fitting models were selected by comparing the Akaike Information Criterion (AIC) and significance test results for main and interaction effects. The relevant factors for each task were entered as follows: 

**Dot-probe task**. For the SAD and control models, trial type (threat, neutral) was entered as a fixed factor and participant as a random factor. For the between-group model trial type and group were entered as fixed factors and participant as a random factor.

**Cueing task**. For the SAD and control models, cue validity (valid, invalid) and cue type (threat, neutral) were entered as fixed factors and participant as a random factor. For the between-group model cue validity, cue type and group were entered as fixed factors and participant as a random factor.

**Irrelevant distractor task**. An initial model assessed effects of irrelevant distractors on RTs. Distractor presence was entered as a fixed factor and participant as a random factor. The other three models included distractor-present trials only. For the SAD and control models, irrelevant distractor type (threat, neutral) and target type (threat, neutral) were entered as fixed factors and participant as a random factor. For the between-group model, target type and group were entered as fixed factors and participant and irrelevant distractor type as random factors.

**Attentional blink task**. For the SAD and control models, lag (1, 2, 3, 4, 5, 6, 7), T1-type (threat, neutral), T2-type (threat, neutral) and distractor-type (threat, neutral) were entered as fixed factors and participant as a random factor. For the between-group model, lag, T1-type, T2-type, distractor-type and group were entered as fixed factors and participant as a random factor. The models revealed no effect of distractor type, which was subsequently dropped from the models. The reported parameter estimates derive from the best fitting model in each case. Additionally, we calculated Bayes factors to assess the strength of the inference for any null results (for completeness, we also added Bayes factors for the tests that turned out to be significant).

## 3. Results

**Figure 2:**
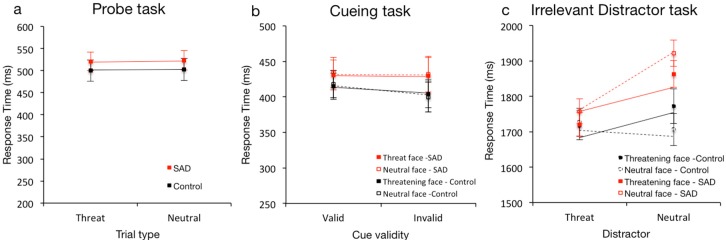
Results for the (**a**) probe, (**b**) cueing and (**c**) irrelevant distractor tasks. The lines represent predictions from the statistical models (see text for details). The error bars show the standard error of the mean (SEM). Note the difference in scales between the panels.

**Figure 3:**
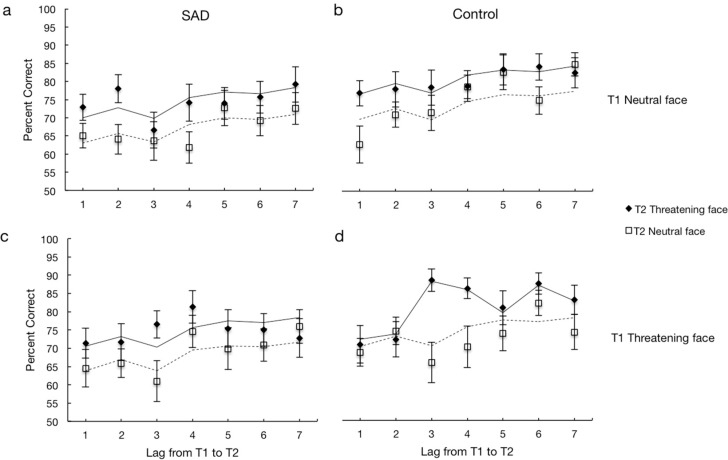
The results for the attentional blink task showing accuracy for T2 when T1 was correct for the SAD group when T1 was neutral (**a**) and when T1 was threatening (**c**), and for the healthy control group when T1 was neutral (**b**) and when T1 was threatening (**d**). Symbols represent the data and the lines represent predictions from the best fitting statistical models (see text for details). Error bars denote SEMs.

In Section 1, we describe how well the tasks differentiate between processing of threatening and neutral faces in a general sense across groups, while in Section 2, we describe how well the tasks measure processing differences between the SAD and control groups. Response times for the dot-probe, cueing and irrelevant distractor tasks are shown in Figure 2 and accuracy for the different conditions of the attentional blink are shown in Figure 3.

### 3.1. Differences in Attentional Processing of Threatening and Neutral Faces

**Dot-probe task**. There were no main effects of trial type (neutral, threat) on RT in either group (SAD: *p* = 0.482, Bayes factor = 1.37, control: *p* > 0.785, Bayes factor = 1.22), demonstrating that the probe task was not sensitive to differences in attentional processing of threatening and neutral faces for either the SAD or control groups.

**Cueing task**. There were no significant main effects of cue type (neutral, threat) on RT for either group (SAD: *p* = 0.643: Bayes factor = 1.27, control: *p* = 0.635; Bayes factor = 1.31). There was a main effect of cue validity (valid, invalid) for the control; b = 12.96, t (64.75) = 2.092, *p* = 0.040; 95% CI = [0.58, 25.34] but not the SAD group (*p* = 0.974; Bayes factor = 1.54). Interactions between cue validity and cue type were not significant for either group (SAD: *p* = 0.920, control: *p* = 0.531; Bayes factor = 1.43). The cueing task was in other words not sensitive to differences in attentional processing for either the SAD or the control groups.

**Irrelevant distractor task**. The irrelevant distractor slowed RTs for both the SAD, b = 200.88, t (32) = 6.440, *p* < 0.001; Bayes factor = 52642; 95% CI = [137.35, 264.42] and control groups, b = 162.29, t (26) = 3.626, *p* = 0.001; Bayes factor = 32.6; 95% CI = [70.30, 254.27]. There was a significant main effect of distractor type in the SAD group with 259 ms faster RTs when the distractor was threatening than neutral, b = −258.82, t (96) = −2.425, *p* = 0.017; Bayes factor = 2.37; 95% CI = [−470.69, −46.95]. The effect of distractor type was not significant for the control group (*p* = 0.367; Bayes factor = 1.32). The main effect of target type was not significant, although numerically, responses were faster for the SAD group for threatening targets (SAD: *p* = 0.072, control: *p* = 0.181; Bayes factor = 1.67). Interactions between distractor and target type were not significant for either group (SAD: *p* = 0.162, control: *p* = 0.232; Bayes factor = 1.7). The irrelevant distractor task therefore revealed some processing differences for threatening versus neutral faces for the SAD but not for the control group.

**Attentional blink task**. There was a main effect of lag for both groups, SAD: F (1, 1559.302) = 2.339, *p* = 0.030; Bayes factor = 2.81; control: F (1, 69.269) = 1439.382, *p* < 0.001; Bayes factor = 5.68 × 10^19^. T2 accuracy was lower for lags 1, 2 and 3 for both groups, indicating an attentional blink that begins to recover at lag 4 (see Figure 3). Most notably, T2 accuracy was higher for both groups when T2s were threatening, 8% higher for the SAD group, b = 0.08, t (1559.292) = 3.780, *p* < 0.001; Bayes factor = 47.6; 95% CI = [0.038, 0.119] and 5% higher for the control group, b = 0.05, t (1355.453) = 2.710, *p* = 0.007; Bayes factor = 4.13; 95% CI = [0.015, 0.094], showing how a threatening T2 is more likely to survive the attentional blink. The attentional blink task is in other words highly sensitive to differences in attentional processing of threatening and neutral faces for both the SAD and control groups (see Figure 3).

### 3.2. Differences in Anxious (SAD) and Healthy (Control) Attentional Processing Patterns of Threatening and Neutral Faces

**Dot-probe task**. Neither the main effect of group (*p* = 0.517; Bayes factor = 1.36), main effect of trial type (*p* = 0.573; Bayes factor = 1.38) nor the interaction between group and trial type were significant (*p* = 0.884; Bayes factor = 1.46) indicating that the dot-probe task was not sensitive to processing differences of threatening and neutral faces between anxious and healthy individuals.

**Cueing task**. Again, neither the main effect of group (*p* = 0.819; Bayes factor = 1.4), Cue Type (*p* = 0.995; Bayes Factor = 1.49), nor Cue Validity (*p* = 0.208; Bayes Factor = 1.08) were significant and neither the interactions between Cue Validity and Cue Type (*p* = 0.608; Bayes Factor = 1.43) nor Cue Validity, Cue Type and Group (*p* = 0.708; Bayes Factor = 1.32) were significant showing no sensitivity to differences between how anxious and healthy individuals process threatening and neutral faces for the cueing task.

**Irrelevant distractor task**. There was a significant main effect of group, b = 767.45, t (171.042) = 2.635, *p* = 0.009; Bayes Factor = 2.95; 95% CI = [192.54, 1342.35] and significant interactions between group and distractor type, b = −362.55, t (142.059) = −2.243, *p* = 0.026; Bayes Factor = 2.97; 95% CI = [−682.05, −43.05] and group and target type, b = −348.65, t (138.938) = −2.169, *p* = 0.032; Bayes Factor = 2.88; 95% CI = [−666.44, −30.86]. Both groups responded faster when both target and distractor were threatening, indicating that participants were vigilant to threat, but they reacted differently to a threatening target when distractors were neutral: the SAD group responded faster but the control group slower. The irrelevant distractor task therefore shows some sensitivity to differences in processing of threatening and neutral faces by group, although the pattern is, admittedly, not very easy to interpret.

**Attentional blink task**. There were both significant main effects of lag, F (6, 2914.687) = 5.316, *p* < 0.001; Bayes Factor = 2080.7 and group, b = 0.07, t (59.518) = 2.243, *p* = 0.029; Bayes Factor = 3.18; 95% CI = [0.01, 0.13]. Figure 3 shows that these results reflect an attentional blink for both groups. Most interesting is the significant interaction between lag, group, T2 type and T1 type, F (6, 2914.840) = 2.145, *p* = 0.046; Bayes Factor = 3.08, revealing differences in anxious and healthy processing of threatening and neutral faces. Figure 3 shows that T2 accuracy differs by whether T1 was neutral or threatening. When T1 was neutral, there was a drop in accuracy at lags 1–3 for both groups (Figure 2a,b), which then recovers at lag 4. Both groups also showed higher accuracy for threatening T2s following neutral T1s. When T1 was threatening, there was a clear difference between the groups (Figure 3c,d). Following a threatening T1, the pattern stayed the same for the SAD group as following neutral T1s; a drop at lags 1–3 and a boost for threatening T2s. Following a threatening T1 there was also a drop in accuracy for the control group at lags 1–2 but the accuracy was not higher for threatening compared to neutral T2s. T2 facial expression, even when threatening, was not preferentially processed for roughly the first 200 ms following a threatening T1, perhaps reflecting that the control group uses more attentional resources to process threatening compared to neutral T1s. At lag 3, accuracy for threatening T2s increases for the control group. To summarize, the attentional blink task reveals a number of interesting patterns. Overall, threatening T1s seem to cause a larger attentional blink than neutral faces, and secondly, threatening T2s are more likely to survive the attentional blink than neutral T2s. There were also interesting differences in performance between the SAD and control groups. Especially notable are the differences in accuracy as a function of temporal position of the threatening stimuli. This aspect of the task may prove particularly useful in answering questions about the nature of attentional biases connected with particular attentional disorders.

## 4. Discussion

Attention is typically drawn towards stimuli with emotional content, and it has been suggested that strong attention biases can play a role in anxiety disorders. We assessed this for threatening versus neutral faces, and for four different attentional tasks in a counterbalanced within-subjects design, involving 33 outpatients with social anxiety disorder and 26 control participants. Our goal was to assess the sensitivity of the different tasks given that the tasks most often used for assessing and treating attentional bias have not shown consistent results (see e.g., [9,13,14,18]). Our results reveal that the attentional blink task shows by far the largest performance difference by facial expression, and that the other tasks show at best small sensitivity to any such differences. Additionally, the attentional blink task revealed intriguing temporal differences in performance between the SAD and control groups suggesting that there are differences in the temporal effects of threatening versus neutral stimuli between the groups.

But our results also show that the two tasks most often used for assessing and modifying attention bias in anxiety disorders (the dot-probe and cueing tasks) failed to detect processing differences of threatening and neutral expressions in both the SAD and the healthy group and did not differentiate between the groups. This is highly important since the lack of sensitivity of these widely used tasks can account for mixed results of studies where these tasks are used to measure or modify attention bias (see e.g., [14]) and low reliability of the dot-probe task [18]. Conversely, in light of our results, the attentional blink task with its unique temporal properties, has to be considered a promising paradigm for investigating attentional shifts to threatening stimuli, and for assessing attentional bias, consistent with our previous results [9]. The attentional blink task proved to be sensitive to differential attentional processing of threat, both within the SAD group and the control group. The irrelevant distractor task then appears to be sensitive to differential processing solely for the SAD group. Additionally, both the attentional blink and irrelevant distractor tasks differentiated between a group with SAD as the primary diagnosis and control participants.

The performance pattern in the attentional blink task is particularly interesting. Most strikingly, threatening T2s survive the attentional blink caused by T1 in all conditions, which highlights the unique sensitivity of the task to differential processing of threat stimuli. Following a threatening first target (T1) in the attentional blink task, processing of the threatening facial expression is undisturbed by a threatening second target (T2) at the first two lags in the control group but not the SAD group. This pattern is reminiscent of “emotion induced blindness” where deficits in processing a target (T2) are larger following an emotionally negative than a neutral picture [3]. While it is perhaps surprising that the attentional blink for threat is weaker for SAD participants while healthy participants show a stronger attentional blink, which may suggest that healthy processing of threat is more thorough than dysfunctional processing of threat among individuals with an anxiety disorder. The results from the irrelevant distractor task are in line with this; when a target is threatening, healthy participants respond more slowly than when it is neutral. The SAD group, on the other hand, responds faster to threatening than neutral targets, which suggests that they have a higher vigilance for threat. These patterns of attending during the attentional blink task deserve further investigation in future studies.

Theoretical accounts of attention bias postulate that it involves either facilitated attention to threat or difficulty disengaging from it and that individuals with vulnerability for anxiety attend preferentially to threat, while healthy individuals will ignore it [31,32] (for review see [33]). Our results suggest that the attentional blink task with its sensitivity to the time course of potential bias, could be used to answer such questions regarding temporal aspects of effects of threatening versus neutral stimuli. Furthermore, our results suggest that attention bias in anxiety disorders may involve hyper-vigilance to perceived threat accompanied by more shallow processing of such information which could contribute to continued overestimation of threat and maintenance of anxiety disorders. The attentional blink task must also be considered a viable option for use in attempts at modifying attentional biases. These are exciting avenues for further study in future experiments. While it is possible that other task combinations with other stimulus sets might lead to different results, it is seems unlikely that any particular aspects of the current stimuli or particular task parameters can explain the large differences that we found between the attentional blink task and the traditionally used tasks.

We finally note that a major strength of our current study was the comprehensive clinical assessment of anxiety, which has been lacking in many studies of attentional biases and may partly explain previous contradictory findings, but also highlights the strong clinical relevance of our current study.

## 5. Conclusions

The attentional blink task, with its intricate temporal patterns, appears to be uniquely suited to assessing temporal aspects of healthy and dysfunctional attentional processing of threatening stimuli. We propose that the next step should be to further develop the attentional blink task for reliable measurement and modification of dysfunctional attentional processing in anxiety disorders.

## 6. Supplementary Information

### 6.1. Clinician Administered Interviews

To assess the clinical status of the participants, the measures listed below were used.

**Mini International Neuropsychiatric Interview**. The MINI [21] is a semi-structured interview assessing Axis I disorders as in *DSM-IV*. The MINI has high reliability and validity in relation to the Structured Clinical Interview for DSM-IV (SCID-IV) with inter-rater reliability ranging from 0.89 to 1.0 [21,22]. The Icelandic version of the MINI has good convergent validity with self-report measures of depression and anxiety symptoms [23]. Here, the inter-rater reliability was high: Percentage of agreement between raters in the healthy control group was 100% for all disorders, and in the SAD group it ranged from 90.9% to 100%.

**The Body Dysmorphic Disorder Diagnostic Module (BDD-DM)**. BDD-DM [24] is a brief semi-structured diagnostic interview for body dysmorphic disorder (BDD). Inter-rater reliability for the Icelandic version of BDD-DM used here was high, with 87.5% percentage agreement between raters in the healthy control group for lifetime BDD and 100% for current BDD, and 100% for lifetime BDD and 90.9% for current BDD for the SAD group.

**Liebowitz Social Anxiety Scale (LSAS)**. LSAS [25] is a semi-structured clinical rating scale designed to measure anxiety and avoidance in 24 social situations. Items are rated on a 0–3 point scale and the total score is comprised of the anxiety and avoidance subscales which gives a measure of symptom severity for SAD. The Icelandic version used here had good internal consistency for the SAD (α = 0.82–0.94) and control groups (α = 0.77–0.90). Additionally, scores on the LSAS predicted a SAD diagnosis on the Icelandic version of the MINI. Inter-rater reliability for the Icelandic version of LSAS was high on both subscales (i.e., the intraclass correlation coefficient [ICC] = 1.00 and 0.90 for anxiety and ICC = 0.91 and 0.94 for avoidance) and for the total score (i.e., ICC = 0.98 and 0.92) for the control and SAD group, respectively.

### 6.2. Self-Report Measures

**The Patient Health Questionnaire-9 (PHQ-9)**. The PHQ-9 [26] measures severity of depressive symptoms during the past 2 weeks using 9 items on a 4 point Likert scale. PHQ-9 has high internal reliability and good test-retest reliability [26]. The Icelandic version had good internal consistency (α = 0.9) in this study.

**Quality of Life Scale (QOLS)**. The QOLS [27] measures quality of life on a seven point Likert scale ranging from 1 (terrible) to 7 (delighted). The Icelandic version has good internal (α = 0.89) and acceptable test-retest reliability (*r* = 0.72) [28]. The QOLS had acceptable internal consistency (α = 0.72) in the current study.

### 6.3. Administration of Clinical Tests and Interviews

Participants in the SAD group were recruited with advertisements seeking people aged 18–65 with social anxiety on social media and at an outpatient anxiety center. Control participants were recruited with advertisements on social media seeking people aged 18–65 not suffering from anxiety or depression. Potential participants for the control group were screened via email to match the SAD group on age and gender. All participants gave informed consent. Trained assessors, licensed psychologists and graduate students in clinical psychology, evaluated all participants with the MINI, LSAS and BDD-DM interviews. Participants in the control group that met criteria for a psychiatric disorder were excluded from the study and referred to appropriate treatment. A licensed clinical psychologist supervised all assessments in weekly meetings where a consensus was reached regarding diagnoses.

Information from questionnaires and interviews was collected on laptop computers using RedCap (Research electronic data capture) [34].

## Figures and Tables

**Table 1:** Clinical characteristics and background variables of the social anxiety disorder (SAD) group and the control group.

	SAD	Control	t/Chi-square (df)	*p*
Age *M (SD)*	27.97 (11.73)	28.88 (9.82)	−0.319 (57)	0.751
Gender (% male)	45.5%	42.3%	0.058 (1)	0.809
Education (% >junior college)	39.4%	69.2%	5.192 (1)	0.023 **
LSAS ^a^	83.27 (20.92)	14.35 (11.06)	15.19 (57)	0.000 ***
PHQ-9 ^b^	10.13 (6.43)	2.12 (2.17)	6.06 (54)	0.000 ***
QOLS ^c^	67.03 (10.85)	91.27 (10.76)	−8.43 (55)	0.000 ***

^a^ Liebowitz Social Anxiety Scale. ^b^ Patient Health Questionnaire–9. ^c^ Quality of Life Scale.** *p* < 0.05. *** *p* < 0.01.
